# "Sleep disparity" in the population: poor sleep quality is strongly associated with poverty and ethnicity

**DOI:** 10.1186/1471-2458-10-475

**Published:** 2010-08-11

**Authors:** Nirav P Patel, Michael A Grandner, Dawei Xie, Charles C Branas, Nalaka Gooneratne

**Affiliations:** 1 Respiratory Specialists and Reading Hospital and Medical Center, Reading, PA, USA; 2 Center for Sleep and Respiratory Neurobiology, University of Pennsylvania, Philadelphia, PA, USA; 3 Division of Sleep Medicine, Department of Medicine, University of Pennsylvania, Philadelphia, PA, USA; 4 Center for Clinical Epidemiology and Biostatistics, University of Pennsylvania, Philadelphia, PA, USA; 5 Division of Geriatric Medicine, Department of Medicine, University of Pennsylvania, Philadelphia, PA, USA

## Abstract

**Background:**

Little is known about the social determinants of sleep attainment. This study examines the relationship of race/ethnicity, socio-economic status (SES) and other factors upon sleep quality.

**Methods:**

A cross-sectional survey of 9,714 randomly selected subjects was used to explore sleep quality obtained by self-report, in relation to socioeconomic factors including poverty, employment status, and education level. The primary outcome was poor sleep quality. Data were collected by the Philadelphia Health Management Corporation.

**Results:**

Significant differences were observed in the outcome for race/ethnicity (African-American and Latino versus White: unadjusted OR = 1.59, 95% CI 1.24-2.05 and OR = 1.65, 95% CI 1.37-1.98, respectively) and income (below poverty threshold, unadjusted OR = 2.84, 95%CI 2.41-3.35). In multivariable modeling, health indicators significantly influenced sleep quality most prominently in poor individuals. After adjusting for socioeconomic factors (education, employment) and health indicators, the association of income and poor sleep quality diminished, but still persisted in poor Whites while it was no longer significant in poor African-Americans (adjusted OR = 1.95, 95% CI 1.47-2.58 versus OR = 1.16, 95% CI 0.87-1.54, respectively). Post-college education (adjusted OR = 0.47, 95% CI 0.31-0.71) protected against poor sleep.

**Conclusions:**

A "sleep disparity" exists in the study population: poor sleep quality is strongly associated with poverty and race. Factors such as employment, education and health status, amongst others, significantly mediated this effect only in poor subjects, suggesting a differential vulnerability to these factors in poor relative to non-poor individuals in the context of sleep quality. Consideration of this could help optimize targeted interventions in certain groups and subsequently reduce the adverse societal effects of poor sleep.

## Background

Sleep is receiving increasing and warranted attention as a health risk factor with a majority of studies showing an association between sleep and mortality [1-4]. However, sparse literature is available on the determinants of sleep in the general population, a topic which can be referred to as "population sleep." Cultural context and socioeconomic determinants, in particular, may play a major role in shaping population sleep practices [5].

Sleep characteristics in the general population has been studied, for the most part, by assessing sleep duration as the outcome [6-14]. Measuring duration is insightful but sleep attainment, in reality, is subject to great degrees of subjectivity when individuals are questioned about whether they receive sufficiently restful sleep. For instance, 6 hours of sleep may have differential physiological, cognitive, and psychosocial effects on different individuals. Furthermore, subjects tend to overestimate self-reported sleep duration when compared against objective measures [15]. We elected to focus on the concept of sleep quality as it theoretically captures broader information including sleep duration, physiologic factors and psychosocial perception of sleep attained. Although the literature examining health outcomes associated with sleep has focused on sleep duration [16-19], there is a growing body of evidence for the role of sleep quality in health outcomes as well [20-24], since sleep duration and quality may exert separate though overlapping effects [25-27].

Lower socioeconomic strata frequently are exposed to a systematically higher risk for poorer health outcomes, morbidity and mortality [28] for a variety of reasons [29]; this has resulted in a socioeconomic health gradient [29]. Differential sleep attainment has been correlated with SES measures [30-32]. Although a majority of investigators have not focused primarily on socioeconomic influences upon sleep or the sample has been too small to meaningfully consider multiple other covariates [6,7,9,13]. Nonetheless, the existing data suggest the interesting concept that disparities in sleep could explain part of the socioeconomic health gradient [31,33].

We hypothesize that socioeconomic factors (income, education level, and employment status) play a significant role in the quality of sleep reported. We elected to explore this in a population-based sample in order to maximize study generalizability. This approach can offer important insights into the effect of socioeconomic disparities on sleep quality, extend our current understanding of sleep attainment in the broader population and help lay the foundation for targeted interventions to improve sleep in different cultural and socio-economic groups.

## Methods

### Subjects

The study dataset comes from a community survey by the Philadelphia Health Management Corporation (PHMC), a non-profit organization that focuses on community health. PHMC administers this uncompensated cross-sectional survey in south east Pennsylvania every two years, with the most recent survey conducted in 2006 [34]. The survey examines health and social well-being of residents in Bucks, Chester, Delaware, Montgomery, and Philadelphia counties. Households are chosen using a random-digit dial methodology [35]. One adult aged greater or equal to 18 years is then selected from each household using the "last birthday" method [35]. Telephone interviews are conducted in English or Spanish and all data are based on subjects' self-reported responses. Permission from the PHMC was obtained before analysis of this dataset.

### Measurements

#### Sleep

Subjects were asked to the answer the following question regarding their sleep quality: "In general, how would you rate the quality of your sleep in the past week on a scale from 1 to 5, with 1 being restless and 5 being restful." This question is similar to question 4 on the Sleep Heart Health Study Morning survey [36].

#### Socioeconomic factors

Income status (represented as being above or below the poverty threshold set by the Census Bureau) was ascertained for each subject based on income [37]. In cases of missing income data, income status was imputed using employment status of the main wage earner, Medicaid insurance status and educational level. Education was based on typical educational benchmarks, with the following response self-reported options: less than high school graduate, high school graduate, some college, college graduate, and post-college level education. Employment status was categorized into employed, unemployed (included disabled), retired, and other (homemaker/student).

### Covariates

Sociodemographic factors included in the analysis were age, sex, race/ethnicity, and marital status. Race/ethnicity was classified into 4 categories: White (not Latino), African-American (not Latino), Latino, and other (Asians, Native American, biracial/mixed ("other" race groups were collapsed into one category due to low rates of endorsement). Marital status categories were married, living with a partner, single, widow, and other (divorce/separated/other).

Health indicators and lifestyle habits considered pertinent to sleep included self-reported health status (poor, fair, good, and excellent), body mass index category [38]. (BMI ≤ 25, 25-30, >30), smoking status (smoking versus non-smoking), heavy alcohol use [39,40] (defined previously by the number of occasions in the preceding 30 days when alcohol consumption exceeded 5 drinks), diagnosis of mental illness (yes or no), and stress levels (low, mild, moderate, high, and very high).

### Statistical analysis

Two procedures in SAS 9.0 (SAS Institute, Inc., Cary, North Carolina) for analyzing survey data, SURVEYFREQ and SURVEYLOGISTIC, were used to perform statistical analysis. All analyses were conducted employing sample weights so that inferences could be made about the target population [41]. As the outcome, sleep quality, is an ordinal variable with 5 levels, we fit a cumulative logit model first. The score chi-square was used for testing the proportional odds assumption. When the proportional odds assumption was not met, we fit a generalized logit model. The final model is a logistic regression model with a dichotomized sleep quality outcome (poor sleep versus good sleep) to facilitate interpretation of such models through odds ratios (OR). The cut-point for poor sleep was determined (sleep quality score = 1) from the results of the generalized logit model. This model revealed that income status, education, or employment (primary exposures) did not demonstrate variable levels of effect upon sleep quality scores as sleep quality progressively increased from 2 to 5. Furthermore, to test the sensitivity of the selection of cut points we employed a poor sleep threshold of greater than or equal to 3 in the same regression models and observed that the direction of effect was similar. We thus elected to use a cut-point of 2 on the 5-point scale to dichotomize sleep quality. This would allow for calculation of odds ratios (OR) that were readily interpretable without obscuring important effects. Logistic regression models taking into account the survey sampling weights were fitted to this dichotomous outcome. A p-value of less than 0.05 was considered statistically significant.

## Results

In 2006, 36,853 potential participants were contacted by PHMC and 10,100 subjects provided responses to the survey. This response rate of 28% is fairly typical for uncompensated telephone surveys [42]. Among the 10,100 respondents, 9,714 (96.2%) answered the sleep question. No information was collected by the surveying agency regarding non-responders. Comparison with Census Bureau's American Community Survey (ACS) 2005 [43] for age, gender, and race/ethnic composition revealed that the PHMC sample was similar to expected population norms. Table 1 provides characteristics of the PHMC sample. Poor sleep was reported by 9.1% of the sample (sleep quality = 1 "restless") and only 30.1% reported a sleep score equal to 5 ("restful").

**Table 1:** Demographic characteristics of PHMC Sample

Demographic Variable		% Total (N = 9,553)	% Males (N = 3,137)	% Females (N = 6,416)
**Age**	***18-39***	26.8	25.1	27.6
	
	***40-64***	50.7	53.4	49.4
	
	***>65***	22.5	21.5	23

**Race**	***White***	65.7	71.1	63.1
	
	***African-American***	21.1	16	23.6
	
	***Latino/Hispanic***	10.6	9.6	11
	
	***Other***	2.6	3.3	2.3

**Income Threshold**	***Above***	73.2	79.5	70.1
	
	***Below***	26.8	20.5	29.9

**Race X Income Categories**	***White, Not Poor***	55.5	62.6	52.0
	
	***White, Poor***	10.2	8.5	11.1
	
	***AA, Not Poor***	11.6	9.7	12.5
	
	***AA, Poor***	9.5	6.4	11.1
	
	***Latino, Not Poor***	4.1	4.6	3.8
	
	***Latino, Poor***	6.5	5	7.2
	
	***Other, Not Poor***	2	2.6	1.7
	
	***Other, Poor***	0.6	0.6	0.6

**Education**	***<High school graduate***	9.4	9	9.6
	
	***High school graduate***	32.1	28.2	33.9
	
	***Some college***	21.3	19.7	22.1
	
	***College graduate***	22.4	24.5	21.3
	
	***Post college***	14.9	18.6	13

**Employment**	***Employed***	59.3	66	56
	
	***Unemployed***	5.1	4.7	5.3
	
	***Retired***	22.4	21.9	22.6
	
	***Disabled***	6.5	5.9	6.8
	
	***Other***	6.7	1.5	9.3

**Marital Status**	***Married***	49	56.5	45.3
	
	***Living with partner***	5	4.7	5.1
	
	***Single***	22.5	23.3	22.1
	
	***Widow***	11.5	5.9	14.2
	
	***Other ***	12	9.5	13.2

**Sleep Quality**	***1 - Restless***	9.6	7.2	10.8
	
	***2***	8.5	8	8.8
	
	***3***	23.9	22.4	24.6
	
	***4***	26.1	29.2	24.6
	
	***5 - Restful***	31.8	33.2	31.2

**General Health**	***Excellent***	31	32.3	30.3
	
	***Good***	47.3	47.4	47.2
	
	***Fair***	16.6	15.7	17
	
	***Poor***	5.2	4.6	5.5

**Mental Illness**	***Absent***	85.5	89.5	83.5
	
	***Present***	14.5	10.5	16.5

**BMI**	***≤25***	39.6	30.4	44.2
	
	***25-30***	35.4	45.7	30.3
	
	***>30***	25	23.9	25.5

**Stress Levels**	***Low (1-2)***	19.6	24.3	17.3
	
	***Mild (3-4)***	20.2	22.9	18.9
	
	***Moderate (5-6)***	27.4	25.8	28.2
	
	***High (7-8)***	20.9	19.5	21.5
	
	***Very high (9-10)***	11.9	7.6	14.1

**Heavy Alcohol Use**	***No***	95.6	92.1	97.3
	
	***Yes***	4.4	7.9	2.7

**Smoking**	***No***	79.3	78.7	79.6
	
	***Yes***	20.7	21.3	20.4

Table 2 summarizes unadjusted odds ratios for poor sleep quality (sleep quality = 1 in the 5 point scale), with and without sample weights. Race/ethnic differences were observed with African-Americans and Latinos reporting poorer sleep compared to Whites (OR = 1.65, 95% CI 1.37-1.98 and OR = 1.59, 95% CI 1.24-2.05, respectively). Income status was strongly associated with poor sleep quality (OR = 2.84 95% CI 2.41-3.35). Each increment in level of education was associated with an increasing protective effect upon sleep quality: post college educational attainment predicted an 82% (95% CI 0.13-0.26) reduction in odds for poor sleep quality compared to subjects with less than high school education levels. Unemployment and a non-married status were significantly associated with poor sleep quality as were poor health status and elevated stress levels. For example, poor health conferred an OR = 8.41 (95% CI = 6.23-11.35) comparing with excellent health and very high stress levels an OR = 4.43 (95% CI = 3.39-5.80) comparing to low stress. Smoking was associated with poor sleep quality whereas alcohol ingestion was not.

**Table 2:** Unadjusted Odds Ratios for Poor Sleep Quality (Sleep Score = 1)

Independent Variables N = 9,533		Weighted			Unweighted		
			
		OR	95% CI	P	OR	95% CI	P
**Age** **(ref = 18-39)**	***40-64***	0.94	0.78 - 1.13	0.5014	0.90	0.77 - 1.06	0.2061
		
	***>65***	0.73	0.58 - 0.92	0.0070	0.79	0.65 - 0.96	0.0185
	
**Sex** **(ref = Male)**	***Female***	1.55	1.30 - 1.86	<0.0001	1.57	1.34 - 1.84	<0.0001
	
**Race** **(ref = White)**	***African-American***	1.65	1.37 - 1.98	<0.0001	1.63	1.38 - 1.91	<0.0001
		
	***Latino/Hispanic***	1.59	1.24 - 2.05	0.0003	1.89	1.55 - 2.31	<0.0001
		
	***Other***	0.85	0.47 - 1.55	0.6017	1.00	0.63 - 1.59	0.9952
	
**Income Threshold** **(ref = Above)**	***Below***	2.84	2.41 - 3.35	<0.0001	2.89	2.52 - 3.32	<0.0001
	
**Education (ref =< HS grad)**	***High school graduate***	0.63	0.49 - 0.81	0.0002	0.61	0.50 - 0.75	<0.0001
		
	***Some college***	0.45	0.34 - 0.59	<0.0001	0.43	0.34 - 0.54	<0.0001
		
	***College graduate***	0.27	0.20 - 0.36	<0.0001	0.28	0.22 - 0.35	<0.0001
		
	***Post college***	0.18	0.13 - 0.26	<0.0001	0.20	0.15 - 0.27	<0.0001
	
**Employment** **(ref = Employed)**	***Unemployed***	2.35	1.72 - 3.20	<0.0001	2.39	1.84 - 3.12	<0.0001
		
	***Retired***	1.05	0.85 - 1.29	0.6561	1.21	1.01 - 1.45	0.0416
		
	***Disabled***	5.05	3.96 - 6.44	<0.0001	4.97	4.06 - 6.09	<0.0001
		
	***Other***	1.55	1.13 - 2.13	0.0060	1.59	1.21 - 2.07	0.0007
	
**Marital Status** **(ref = Married)**	***Living with partner***	2.15	1.55 - 2.98	<0.0001	2.26	1.70 - 2.99	<0.0001
		
	***Single***	1.70	1.38 - 2.08	<0.0001	1.85	1.55 - 2.19	<0.0001
		
	***Widow***	2.14	1.68 - 2.72	<0.0001	1.98	1.60 - 2.44	<0.0001
		
	***Other ***	2.04	1.58 - 2.65	<0.0001	1.74	1.41 - 2.16	<0.0001
	
**General Health (ref = Excellent)**	***Good***	1.61	1.28 - 2.03	0.0001	1.65	1.35 - 2.02	<0.0001
		
	***Fair***	4.00	3.12 - 5.14	<0.0001	4.16	3.37 - 5.15	<0.0001
		
	***Poor***	8.41	6.23 - 11.35	<0.0001	8.96	6.95 - 11.55	<0.0001
	
**Mental Illness** **(ref = Absent)**	***Present***	2.47	2.04 - 3.00	<0.0001	2.31	1.97 - 2.71	<0.0001
	
**BMI** **(ref =≤ 25)**	***25-30***	0.97	0.79 - 1.17	0.7300	1.01	0.86 - 1.19	0.8980
		
	***>30***	1.53	1.26 - 1.87	<0.0001	1.52	1.29 - 1.80	<0.0001
	
**Stress Levels** **(ref = Low(1-2))**	***Mild (3-4)***	0.84	0.62 - 1.14	0.2753	0.92	0.71 - 1.19	0.5256
		
	***Moderate (5-6)***	0.92	0.69 - 1.21	0.5446	1.08	0.86 - 1.37	0.5052
		
	***High (7-8)***	1.39	1.05 - 1.84	0.0213	1.53	1.21 - 1.93	0.0004
		
	***Very high (9-10)***	4.43	3.39 - 5.80	<0.0001	4.8	3.83 - 6.01	<0.0001
	
**Heavy Alcohol Use (ref = No)**	***Yes***	1.04	0.72 - 1.49	0.8500	1.19	0.87 - 1.63	0.2724
	
**Smoking (ref = No)**	***Yes***	2.05	1.72 - 2.45	<0.0001	1.96	1.69 - 2.28	<0.0001

Table 3 shows unweighted results and Table 4 shows weighted results from logistic regression with poor sleep as the dependent variable; the results from the weighted and unweighted analyses are generally similar. In both weighted and unweighted analyses, three models were constructed; all three models were adjusted for age and sex. Model 1 shows the race-specific effects of income status on sleep quality. The data was analyzed in this manner as we observed significant interaction (p < 0.0001) between income status and race for Whites, African-Americans and Latinos; by subsequently creating categories of race-income status, we were able to derive ORs for poor sleep for each category that facilitate interpretation of study findings. Model 1 compares sleep quality amongst different race-income status groups to not poor Whites (referent group): Minority race/ethnic groups demonstrated increased odds for poor sleep. African-Americans and Latinos below the poverty line especially demonstrated significantly increased likelihood of poor sleep (OR = 2.72, 95% CI 2.13-3.46 and OR = 2.57, 95% CI 1.88-3.51 respectively). However, impoverished Whites had the highest likelihood of poor sleep (OR = 4.20 95% CI 3.30-5.35).

**Table 3:** Odds Ratios (OR) and 95% Confidence Intervals (CI) from Multivariable Logistic Regression for Sleep Quality, Adjusted for Age and Sex (Unweighted)

Independent Variables N = 9,533		Model 1		Model 2		Model 3	
				
		OR	95% CI	OR	95% CI	OR	95% CI
**Race -Income** **(ref = White, Not Poor)**	***White, Poor***	3.64**	2.98 - 4.45	2.19**	1.76 - 2.72	1.79**	1.43 - 2.24
			
	***AA, Not Poor***	1.50*	1.19 - 1.90	1.25	0.98 - 1.59	1.25	0.97 - 1.60
			
	***AA, Poor***	2.89**	2.34 - 3.57	1.51*	1.18 - 1.92	1.25	0.97 - 1.61
			
	***Latino, Not Poor***	1.45	1.00 - 2.10	1.23	0.85 - 1.79	1.20	0.81 - 1.76
			
	***Latino, Poor***	3.07**	2.41 - 3.90	1.48*	1.11 - 1.96	1.23	0.92 - 1.65
			
	***Other, Not Poor***	1.12	0.63 - 1.99	1.12	0.63 - 2.00	1.11	0.61 - 2.01
			
	***Other, Poor***	2.08	0.93 - 4.62	1.13	0.50 - 2.59	0.95	0.40 - 2.22
		
**Education (ref =< HS grad)**	***High school graduate***	-	-	0.84	0.67 - 1.04	0.94	0.75 - 1.18
			
	***Some college***	-	-	0.63*	0.49 - 0.81	0.74*	0.57 - 0.96
			
	***College graduate***	-	-	0.50**	0.37 - 0.66	0.66*	0.49 - 0.89
			
	***Post college***	-	-	0.42**	0.30 - 0.58	0.57*	0.40 - 0.81
		
**Employment** **(ref = Employed)**	***Unemployed***	-	-	1.62*	1.23 - 2.14	1.28	0.96 - 1.70
			
	***Retired***	-	-	1.18	0.90 - 1.53	1.03	0.78 - 1.36
			
	***Disabled***	-	-	3.12**	2.48 - 3.91	1.70**	1.32 - 2.19
			
	***Other***	-	-	1.21	0.92 - 1.61	1.16	0.87 - 1.56
		
**Marital Status** **(ref = Married)**	***Living with partner***	-	-	1.64*	1.22 - 2.20	1.46*	1.07 - 1.99
			
	***Single***	-	-	1.20	0.99 - 1.45	1.12	0.92 - 1.36
			
	***Widow***	-	-	1.58*	1.23 - 2.03	1.48*	1.14 - 1.91
			
	***Other ***	-	-	1.16	0.92 - 1.46	0.98	0.77 - 1.24
		
**General Health (ref = Excellent)**	***Good***	-	-	-	-	1.31*	1.06 - 1.62
			
	***Fair***	-	-	-	-	2.38**	1.87 - 3.03
			
	***Poor***	-	-	-	-	3.68**	2.71 - 4.98
		
**Mental Illness** **(ref = Absent)**	***Present***	-	-	-	-	1.12	0.92 - 1.35
		
**BMI** **(ref =≤ 25)**	***25-30***	-	-	-	-	1.06	0.89 - 1.27
			
	***>30***	-	-	-	-	1.2	1.00 - 1.44
		
**Stress Levels** **(ref = Low(1-2))**	***Mild (3-4)***	-	-	-	-	1.04	0.79 - 1.35
			
	***Moderate (5-6)***	-	-	-	-	1.11	0.86 - 1.41
			
	***High (7-8)***	-	-	-	-	1.45*	1.13 - 1.87
			
	***Very high (9-10)***	-	-	-	-	3.04**	2.36 - 3.91
		
**Heavy Alcohol Use (ref = No)**	***Yes***	-	-	-	-	1.18	0.84 - 1.65
		
**Smoking (ref = No)**	***Yes***	-	-	-	-	1.28*	1.08 - 1.51

* = p < .05

** p < .0001

**Table 4:** Odds Ratios (OR) and 95% Confidence Intervals (CI) from Multivariable Logistic Regression for Sleep Quality, Adjusted for Age and Sex (Weighted)

Independent Variables N = 9,533		Model 1		Model 2		Model 3	
				
		OR	95% CI	OR	95% CI	OR	95% CI
**Race -Income** **(ref = White, Not Poor)**	***White, Poor***	4.20**	3.30 - 5.35	2.46**	1.87 - 3.23	1.95**	1.47 - 2.58
			
	***AA, Not Poor***	1.75**	1.34 - 2.29	1.45*	1.09 - 1.93	1.45*	1.08 - 1.94
			
	***AA, Poor***	2.72**	2.13 - 3.46	1.40*	1.07 - 1.85	1.16	0.87 - 1.54
			
	***Latino, Not Poor***	1.51	0.97 - 2.35	1.29	0.83 - 2.00	1.33	0.83 - 2.11
			
	***Latino, Poor***	2.57**	1.88 - 3.51	1.24	0.86 - 1.80	1.05	0.72 - 1.52
			
	***Other, Not Poor***	1.05	0.50 - 2.17	1.06	0.51 - 2.23	1.01	0.48 - 2.14
			
	***Other, Poor***	1.57	0.57 - 4.34	0.87	0.29 - 2.62	0.67	0.19 - 2.30
		
**Education (ref =< HS grad)**	***High school graduate***	-	-	0.79	0.60 - 1.04	0.87	0.66 - 1.15
			
	***Some college***	-	-	0.59*	0.43 - 0.81	0.67*	0.48 - 0.93
			
	***College graduate***	-	-	0.43**	0.30 - 0.61	0.55*	0.38 - 0.79
			
	***Post college***	-	-	0.35**	0.23 - 0.53	0.47*	0.31 - 0.71
		
**Employment** **(ref = Employed)**	***Unemployed***	-	-	1.62*	1.18 - 2.21	1.31	0.95 - 1.82
			
	***Retired***	-	-	1.04	0.75 - 1.43	0.92	0.66 - 1.28
			
	***Disabled***	-	-	3.16**	2.40 - 4.17	1.68*	1.24 - 2.29
			
	***Other***		-	1.16	0.84 - 1.61	1.17	0.83 - 1.65
		
**Marital Status** **(ref = Married)**	***Living with partner***	-	-	1.51*	1.06 - 2.15	1.35	0.94 - 1.95
			
	***Single***	-	-	1.03	0.82 - 1.31	0.98	0.77 - 1.24
			
	***Widow***	-	-	1.75*	1.30 - 2.36	1.63*	1.20 - 2.22
			
	***Other ***	-	-	1.28	0.97 - 1.69	1.06	0.80 - 1.40
		
**General Health (ref = Excellent)**	***Good***	-	-	-	-	1.26	1.00 - 1.61
			
	***Fair***	-	-	-	-	2.28**	1.71 - 3.03
			
	***Poor***	-	-	-	-	3.48**	2.39 - 5.07
		
**Mental Illness** **(ref = Absent)**	***Present***	-	-	-	-	1.2	0.96 - 1.52
		
**BMI** **(ref =≤ 25)**	***25-30***	-	-	-	-	1.04	0.84 - 1.29
			
	***>30***	-	-	-	-	1.23	0.99 - 1.53
		
**Stress Levels** **(ref = Low(1-2))**	***Mild (3-4)***	-	-	-	-	0.93	0.68 - 1.28
			
	***Moderate (5-6)***	-	-	-	-	0.91	0.68 - 1.23
			
	***High (7-8)***	-	-	-	-	1.24	0.92 - 1.67
			
	***Very high (9-10)***	-	-	-	-	2.74**	2.03 - 3.71
		
**Heavy Alcohol Use (ref = No)**	***Yes***	-	-	-	-	0.99	0.66 - 1.48
		
**Smoking (ref = No)**	***Yes***	-	-	-	-	1.27*	1.04 - 1.56

* = p < .05

** p < .0001

After including education, employment and marital status (Model 2), we observed an attenuated but persistent "sleep disadvantage" (worse sleep quality score) amongst impoverished groups especially in Whites (OR = 2.46, 95% CI 1.87-3.23). Model 3, the final model, included several health indicators considered to be relevant to sleep quality. The association between impoverished Whites and poor sleep remained highly significant (OR = 1.95, 95% CI 1.47-2.58). However, African-Americans below the poverty line no longer demonstrated increased odds for poor sleep when these additional factors were included, whereas the increased odds of poor sleep quality in not poor African-Americans persisted with minimal attenuation (OR = 1.45, 95% CI 1.08-1.94). Additional SES factors that remained significantly associated with poor sleep quality in Model 3 included lower levels of education and unemployment. Having at least a college level education (compared to less than high school education) was associated with approximately a 50% reduction in odds for poor sleep. Being widowed or living with a partner (compared to married) was associated with increased odds of poor sleep. Fair or poor self-rated health (compared to excellent health) was significantly associated with poor sleep. Very high levels of stress and smoking were also significant predictors of poor sleep. Though not significant, a trend in association was observed for obesity (BMI > 30) and psychiatric diagnosis. Taken together, of these additional factors included into Model 3, self-reported general health and stress level had the largest effects on increasing the risk of poor sleep amongst impoverished individuals. In contrast, for minority subjects above the poverty line, these employment, education, general health and marital status factors had modest effect on the increased likelihood of poor sleep.

## Discussion

The public health ramifications of sleep are far-reaching and under-recognized as stated in a recent report by the Institute of Medicine [44]. Many have reported the ill effects that poor sleep, defined by suboptimal sleep duration or the presence of sleep disorders, confers upon health (including mortality) [1-4,45], well-being, and society [44]. The collective economic impact of impaired/restricted sleep is enormous and has been conservatively estimated at $107 billion [44]. It is, therefore, critical to advance our understanding of the determinants of sleep attainment given the impact.

This study included and adjusted for several covariates that plausibly influence sleep quality that have not been addressed in prior studies [6,8,11-13], with interesting results. A significant "sleep disparity" exists in the population sample, with African-American and Latino groups overall having poorer sleep quality than the White, non-poor, referent group. Amongst these minority subjects, the impoverished subgroups reported the highest odds for poor sleep. The inclusion of other socio-economic and health covariates, however, significantly modified these findings. Of particular interest, health covariates markedly attenuated the relationship between poor sleep and race/ethnicity for impoverished individuals: impoverished African-American subjects demonstrated a diminution of the odds ratio for poor sleep upon including socio-economic covariates such as employment and education (model 2), but the odds ratio became non-significant only when health covariates were included (model 3). This offers insights into potential targets for intervention by suggesting that minorities are particularly vulnerable to the effects of poor health on sleep quality.

These results are consistent with literature linking SES [6,9,12,33,46-49] and race/ethnicity disparities to sleep attainment [7,9,50]. However, our analysis advances on these studies by reporting for the first time the interactive effects of race and SES upon sleep quality in a large population sample. There is a growing appreciation that race and SES may operate in an interactive manner on health outcomes [51]. Thus, instead of the issue being an issue of race or class, it may be race and class [52]. This may be true for sleep attainment and the associated health outcomes.

Our observation that impoverished White subjects demonstrated the highest odds for poor sleep in contrast to other race/ethnic groups who did not have increased likelihood of poor sleep after adjusting for the same covariates is intriguing when examined in the context of existing literature on health disparities- typically poor minority groups experience the most health disadvantage [53]. We believe this adds new perspective to the minority poverty hypothesis that refers to the unique disadvantage experienced by impoverished African-Americans [51]. The underlying mechanism of poorer reported sleep quality in this group is unclear and may represent biological, psychosocial, cultural and environmental processes. It is possible, for example, that "expectations" of sleep quality led to a greater sense of impaired sleep quality in Whites. This warrants further investigating to assist in future public health campaigns and sleep-health policy.

African-Americans above the poverty line had significantly increased odds for poor sleep compared to the White referent group and African-Americans below the poverty line in multivariable adjusted analysis. This raises further questions: 1) What are the explanations for sleep quality disparity amongst not poor African-Americans and Whites? This may be related to societal structure and/or due to a differential SES/sleep relationship in African-Americans and Whites [53] and; 2) Why do African-Americans above the poverty line have increased likelihood of poor sleep than African-Americans below the poverty line? Sleep disadvantage in the latter group, as noted earlier, may be explained by education, employment and health factors. In African-Americans above the poverty line, though, their higher SES has been hypothesized to foster positive social, psychological, and economic skills that shield against the effect of adversity [54] which in theory would protect sleep attainment. The persistence of poor sleep in the fully adjusted Model 3 analysis in non-poor African-Americans may instead be related to other factors such as higher sleep/health expectations, career demands, and social roles [5]. As noted earlier, though, this same finding did not hold true for Whites: Impoverished Whites demonstrated worse sleep quality than non-impoverished Whites even in adjusted analysis. This raises the intriguing question of whether poverty has differential effects on symptom perception in different race/ethnic groups even after adjusting for SES and health factors. Several explanations in a variety of socio-ecological domains may be posited for this sleep-race-SES gradient: differences in health behavior (for example, self-efficacy, perception, attitudes and value expectancy), psychosocial circumstances, and environment (social and physical) can disparately affect sleep. Future research relying on qualitative methods and grounded in health behavior theory could add to our understanding of these factors. Of note, this finding of a statistically significant elevated OR for impaired sleep in not poor African-Americans even in multivariate modeling that was observed in the weighted model was not as prominent in the unweighted model: for the unweighted model, there remained an elevated OR (1.25), but the lower limit of the confidence interval was 0.97, thus it represented a trend towards statistical significance in the unweighted model. Thus, the findings were similar in the weighted and unweighted models.

The literature linking sleep and health continues to grow. Our study is consistent with this as we observed that poor health was associated with an almost 4-fold increased likelihood of poor sleep in the final model and represented one of the most significant factors (Model 3). It is important to note that the relationship between health and sleep quality is likely bidirectional and/or parallel: sleep can influence health and vice-versa.

Assessing sleep quality over sleep quantity may have several advantages. We believe that assessing sleep quality captures multiple domains of sleep including quantity, consolidation, daytime functioning, and sleep satisfaction. Sleep quality, compared with sleep quantity, has superior relation to measures of health, well-being, and sleepiness [55]. Sleep duration studies typically define a referent duration as an optimal. This can overlook sleep sufficiency, disruption and of course known biological differences in sleep requirements [56]. Finally, there is a reported discrepancy between subjective and objective sleep duration [15]. Theoretically, inquiry about sleep quality may encompass these concerns.

### Limitations

Respondents were from Philadelphia metropolitan area thereby limiting generalizability to similar locales. Nonetheless, the respondents were from diverse race/ethnic backgrounds. Telephone surveys are inherently limited by coverage (low socioeconomic status, disabled, and institutionalized subjects may not have access to a telephone) and response rate. A response rate of 28% is consistent with reported attrition discussed in the literature [57] and does not necessarily translate to non-response bias [58]. However, this is still a low response rate by absolute standards, and may reflect a number of biases in the sample, systematically excluding those who cannot be on the phone for longer periods of time and/or are not willing or able to participate in research and reflecting biases caused by social desirability and/or demand characteristics. Importantly, this study's sample characteristics are generally similar to Census data for the same geographical area. In addition, we have presented both weighted (correcting for differences relative to Census data) and unweighted data; the results are fairly similar for both methods.

The cross-sectional design limits inference of causality in the effect of SES on sleep quality. SES measurement is challenging and not completely captured by any single or combination of factors [59]. Nonetheless, all measured SES dimensions (income status, low education level, and unemployment) were significantly associated with poor sleep. Furthermore, we recognize that different socioeconomic factors may affect health at different times in the lifespan [59].

## Conclusions

Our study provides insight into the complex relationship of socioeconomic factors, race and health on population sleep, a major public health issue [44]. While we observed highly significant race-socioeconomic differences in sleep quality in a relatively large population sample, these effects were different in various race/ethnic groups. Minority groups had worse overall sleep quality than Whites, but when examining poor subjects in various race/ethnic subgroups, we observed that impoverished Whites had paradoxically worse sleep than corresponding impoverished minority groups. Furthermore, this poor sleep quality was significantly mediated by education, employment and health factors in poor individuals, but not in individuals above the poverty line. This highlights the differential impact of SES and general health factors in poor minority groups.

Future research evaluating for SES disparities in sleep is an important exercise to: 1) highlight inequality in an overlooked but important health behavior; 2) to begin to understand how sleep attainment is influenced and; 3) to be able to target higher-risk groups for interventions. The impact of sleep behavior on health outcomes is a rapidly growing literature as is our understanding of social inequalities in health. Thus, disparity in sleep quality as an explanation of socioeconomic inequality in health is an area that also requires further attention.

## Competing interests

The authors declare that they have no competing interests.

## Authors' contributions

NP and NG conceived of and designed the study. NP carried out the majority of study components. DX and CB directed data analysis and interpretation. NP, MG and NG helped to draft the manuscript. All authors have read and approved the final manuscript.

## Pre-publication history

The pre-publication history for this paper can be accessed here:

http://www.biomedcentral.com/1471-2458/10/475/prepub
